# Remineralization of early enamel lesions with a novel prepared tricalcium silicate paste

**DOI:** 10.1038/s41598-022-13608-0

**Published:** 2022-06-15

**Authors:** Kareem Hamdi, Hamdi H. Hamama, Amira Motawea, Amr Fawzy, Salah Hasab Mahmoud

**Affiliations:** 1 Operative Department, Faculty of Dentistry, Zagazig University, Zagazig, Egypt; 2 Operative Dentistry Department, Faculty of Dentistry, Mansoura University, Algomhoria St, Mansoura City, 35516 Egypt; 3 Department of Pharmaceutics, Faculty of Pharmacy, Mansoura University, Mansoura, Egypt; 4 UWA Dental School, University of Western Australia, Perth, Australia

**Keywords:** Biological techniques, Diseases, Health care, Medical research, Risk factors, Signs and symptoms

## Abstract

To evaluate the remineralization potential of prepared tricalcium silicate (TCS) paste compared to silver diamine fluoride-potassium iodide (SDF-KI) and casein phosphopeptide-amorphous calcium phosphate (CPP-ACP) on artificial enamel lesions. Thirty permanent sound molars were collected for the study. After cleaning, root cutting, and applying acid-resistant nail varnish, leaving a 4 × 4 mm buccal window, the teeth were subjected to demineralization process. The teeth were divided into three treatment groups (n = 10). In each group, the teeth were sectioned buccolingually to obtain two halves (30 self-control and 30 experimental halves). The self-control halves were subjected to cross-sectional microhardness (CSMH), energy-dispersive X-ray spectroscopy at 50, 100, and 150 µm from the external enamel surface, and micromorphological analysis at the superficial enamel surface. The experimental halves were subjected to the same tests after 30 days of remineralization. Three-way analysis of variance (ANOVA) outcomes showed no significant difference in CSMH after treatment among the three different groups at the different levels (p > 0.05). Meanwhile, three-way ANOVA outcomes showed a significant difference in calcium/ phosphate ratio after treatment among the three different groups at the different levels. (p < 0.05). The tricalcium silicate paste used in this study showed potential remineralization in subsurface enamel lesions.

## Introduction

The first clinical sign of enamel demineralization is a white spot lesion (WSL). The demineralization process becomes confined within the superficial enamel layer leading to marked porosity. According to the International Caries Detection and Assessment System (ICDAS), WSLs can be categorized into scores 1 and 2. ICDAS score 1 is characterized by the appearance of WSLs after a relatively prolonged air surface drying. In ICDAS score 2, WSLs are obvious in dry and wet conditions^1^. In both scores, the structural integrity of the enamel is maintained and intact with no localized breakdown. When these lesions are left untreated, they might progress to a continuous loss of minerals and break down until cavitation^2^. Current cariology research focused on the effectiveness of the application of some remineralizing agents that maintained ion supersaturation in the oral environment surrounding these lesions. This might enhance calcium and phosphate ions to fill the formed microspores and stop further mineral loss^3^.

Fluoride has been considered the most popular remineralizing agent in the past decades. Fluoride can inhibit plaque biofilm and enhance calcium and phosphate ion precipitation within the biofilm^4^. Several laboratory studies have assumed that fluoride ions can replace the –OH group in the hydroxyapatite (HAp)-forming fluorapatite, which has superior resistance to acidic attacks^5^. The medical treatment of early enamel carious lesions encompassed the use of fluoride-based remineralizing agents, such as sodium fluoride and stannous fluoride^6^. Recently, silver diamine fluoride (SDF) has gained wide acceptance in the prevention and treatment of these early enamel lesions^6^. In 2014, the U.S. Food and Drug Administration approved SDF as caries arrest therapy for adults and children^6^. The carries arrest ability of SDF has shown marked superiority over other fluoride-based products, such as sodium fluoride and stannous fluoride^7,8^. In addition to its superior role in caries prevention and arrest, several laboratory studies have also assessed its remineralization power^9,10^.

Nowadays, non-fluoride (bioavailable calcium phosphate) remineralizing agents are considered the gold standard medical treatment for early enamel carious lesions. This category of bioactive remineralizing agents includes amorphous calcium phosphate (ACP), casein phosphopeptide (CPP-ACP), and tricalcium phosphate^11^.

CPP-ACP is phosphorylated casein extracted from milk. Its remineralization power is ascribed to the chemical effect of phosphorylated casein in addition to the presence of calcium and phosphate ions^12^. At low pH, ACP separates from CPP, which usually increases the salivary saturation with Ca^2+^ and PO_4_^3−^ ions. After this separation, CPP can stabilize ACP in the oral biofilm, providing an amorphous state of supersaturation to maintain alkaline pH and enhance the remineralization process^13,14^.

Calcium silicate-based materials (β-CaSiO_3_, β-Ca_2_SiO_4_, and Ca_3_SiO_4_) play an important role in hard tissue regeneration. It shows good bioactivity, biocompatibility, and the ability to induce bone-like appetite formation. Most literature about these materials highlights their reparative role in restorative and endodontic fields. Current scientific literature shows little evidence suggesting using these materials in the medical management of early enamel lesions. When tricalcium silicate (TCS; Ca_3_SiO_4_) comes into contact with saliva, it dissolves and deposits the silanol group (Si–O) on the enamel surface^15^. This functional group can bind to Ca^+^ ions and induce HAp precipitation. Furthermore, the salivary phosphate group (PO_4_^3−^) can attract Ca^2+^ ions through the silanol group, forming a calcium phosphate (Ca–P)-rich layer that plays a great role in enamel remineralization^15^. Another in vitro study has emphasized the remineralization effect of combined TCS with fluoride on demineralized enamel^16^. TCS was used in the slurry form in the literature^15,16^.

Considering the limited evidence suggesting the remineralization of early enamel lesions using TCS, this study was designed to evaluate the remineralization power of TCS. TCS was laboratory prepared in a novel paste formula, unlike the slurry formula incorporated in previous studies. The tested null hypothesis was that there was no significant difference among the prepared TCS, CPP-ACP, and SDF-potassium iodide (SDF-KI) in the remineralization of subsurface enamel lesions at 50, 100, and 150 µm.

## Methods

Materials used in the study according to the manufacturer’s recommendations are listed in Table1.

**Table 1:** Materials used in the study.

Material	Composition	Manufacture
Riva star	• Silver capsule: containing silver fluoride • Green capsule: containing KI	SDI Ltd., Bayswater, VIC 3153 Australia
Tooth mousse	CPP-ACP	GC Europe N.V
TCS paste	• 5% (w/v) TCS • 2.5% (w/v) gelatin • 2.5% (w/v) carboxymethyl cellulose (CMC) • 2% (w/v) propylene glycol • 0.1% (w/v) methylparaben • Distilled water	Prepared at the Department of Pharmaceutics, Faculty of Pharmacy, Mansoura University

Thirty permanent caries-free human molars were collected for the study. The molars were collected from Oral and Maxillofacial Surgery Department in Faculty of Dentistry, Mansoura University. The protocol of teeth collection and storage was approved by the Mansoura University Faculty of Dentistry ethical committee (approval no. M03060819). Patients who voluntarily donated their “waste” extracted teeth provided informed consent to collect the teeth for the experimental study. The collected teeth were rinsed with deionized water and cleaned with periodontal curettes to remove any soft-tissue debris. The teeth were stored in 0.1% thymol solution and refrigerated at 4 °C until use. The teeth were checked visually using an LED light and magnifying loupes × 3.5 (Amtech, America) to detect the presence of any enamel surface defects or microcracks. Teeth with buccal surface cracks or stains were excluded from the study.

Thirty molars were subjected to the demineralization protocol, which will be discussed in detail in the following section. The demineralized specimens were divided into three groups (n = 10) according to the applied remineralizing agent. The demineralized specimens were treated with SDF-KI (Riva Star; Group A), CPP-ACP cream (GC Tooth Mousse; Group B), and prepared TCS paste (Group C).

### Preparation of artificial carious lesions

To create enamel surface lesions resembling naturally developed WSLs, the buccal surface of the teeth was polished with 600, 800, and 1200 grit paper (Fuji Star, Sankyo Rikagaku, Saitama, Japan) consecutively under running water to remove a prismatic enamel layer. The buccal surface was covered with adhesive tape (4 × 4 mm in size), and acid-resistant nail varnish was used to cover the whole surface of each tooth. The tape was removed, leaving a 4 × 4 mm window on the buccal surface. The extracted teeth were immersed in a demineralizing solution (demineralizing solution composed of 2.2 mM calcium chloride, 2.2 mM sodium phosphate, and 0.05 M acetic acid with 1 M potassium hydroxide to obtain a pH of 4.4) for 72 h at 37 °C^17^. The demineralizing solution was changed every 24 h to keep the pH constant. The 30 demineralized molars were cut carefully at the level of the cementoenamel junction to keep the crowns only using a low-speed diamond saw (Isomet; Buehler, Lake Bluff, IL, USA). The crowns were fixed in acrylic blocks then sectioned vertically under running water in the buccolingual direction using a low-speed diamond saw (Isomet; Buehler). Two halves were present. One was used as a self-control specimen, and the other was used as an experimental one.

After obtaining 60 halves of the demineralized specimens, the internal surface of the specimens that would be subjected to a cross-sectional microhardness (CSMH) test was abraded using silicon carbide paper and lapped with a cloth. The silicon carbide paper was attached to a rotary electric polishing machine (Ecomet 250; Buehler). The used silicon carbide polishing papers were changed after every five specimens^18^. The 30 halves of the self-control specimens were fixed in acrylic blocks with their buccal or lingual surface embedded in acrylic blocks and their internal surface facing upward. The self-control halves were kept in deionized water until they were tested. The other 30 experimental halves were subjected to the treatment protocol that will be discussed thereafter.

### Preparation of the TCS paste

The optimized gel was prepared according to the following protocol. First, the gelatin solution was prepared by dissolving 2.5% (w/v) gelatin in hot distilled water, and a magnetic stirrer was employed at 200 rpm and 70 ℃ for 30 min until complete dissolution (hot plate and magnetic stirrer; Misung Scientific Co., Korea). Simultaneously, a 2.5% (w/v) CMC solution was prepared by heating its aqueous solution to 60 °C to 70 °C in distilled water with stirring at 200 rpm using a magnetic stirrer. Sequential polymerization was induced through electrostatic interaction by adding CMC solution to the gelatin solution dropwise under magnetic stirring for 15 min at 70 °C and 200 rpm to obtain a homogenized hydrogel^19^. The solution was left overnight at room temperature to ensure complete mixing and gel formation. A 5 g TCS powder with a particle size of 1 to 10 µm was levigated with propylene glycol (2 mL). The levigated 5% (w/v; maximum dose) TCS was added slowly and mixed to this hydrogel using a homogenizer (Heidolph, Germany). Methylparaben (0.1%, w/v) was used as a preservative. A few drops of 1 N HCl were added to adjust the pH to the desired level (pH 6.8) and maintain the viscosity of the paste. The remaining amount of water was added with gentle stirring at room temperature to prevent the formation of air bubbles until paste formation. The pH of the paste was monitored using a pH probe (Denver Instrument Ub-10 Bio Kit, 115 VAC, USA). Finally, the prepared paste was filled in a collapsible aluminum tube until use.

### Surface treatment methods

The internal surface (cross-section) of the experimental halves was covered with an adhesive tape to ensure that the treatment was subjected only to the external enamel surface. The experimental halves in Group A were painted buccally through the window by a micro brush with SDF-KI according to the manufacturer’s instructions (only one time as a professional application). Specimens in Group B were painted buccally through the window by a micro brush with CPP-ACP according to the manufacturer’s instructions (twice daily). The cream was left on the superficial buccal surface for 3 min, and the specimens were washed with deionized water for 5 s. Specimens in Group C were painted buccally through the window by a micro brush with the prepared TCS paste (twice daily). The paste was left on the superficial buccal surface for 3 min, and the specimens were washed with deionized water for 5 s. The experimental halves were stored in artificial saliva during the remineralization period (30 days) and kept in an incubator at 37 °C. The composition of artificial saliva was as follows: methyl-p-hydroxybenzoate (2 g/L), sodium CMC (10 g/L), CaCl_2_⋅2H_2_O (0.166 g/L), KH_2_PO_4_ (0.3 g/L), K_2_HPO_4_ (0.8 g/L), and KCl (0.6 g/L) adjusted to pH 6.8^20^. Artificial saliva was replaced with fresh artificial saliva every 24 h to maintain the pH constant^21^. After completion of the remineralization period, the experimental halves were subjected to the same tests as the self-control halves.

### CSMH test for self-control specimens

**Figure 1:**
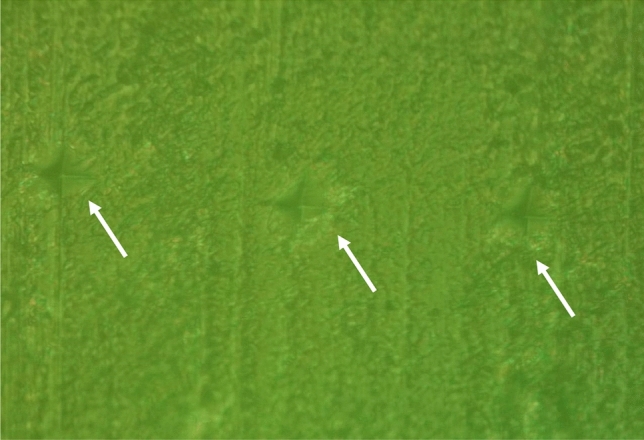
Microscopic image from a microhardness optical microscope (× 40) showing the power of microindentations at three different points (50, 100, and 150 µm) from the external enamel surface (white arrows).

Vickers CSMH test was applied with a force of 100 g for 10 s (Wilson® Tukon 1102/1202 Series; Buehler) at three different points (50, 100, and 150 µm; at the middle third) from the external enamel surface. These three indentations were determined and measured from the external enamel surface via the microhardness microscope annotation tool (Fig. 1).

### Micro-Raman spectroscopy analysis

**Figure 2:**
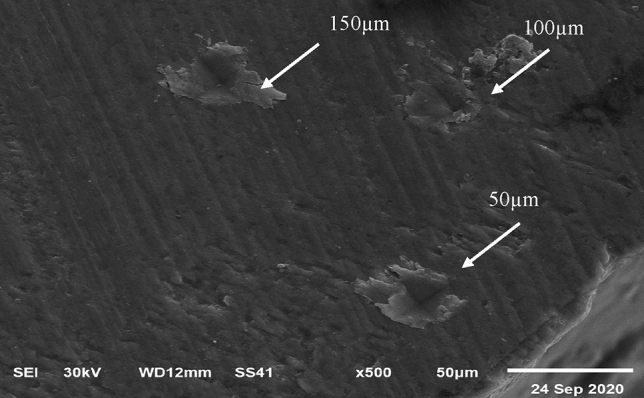
SEM micrograph showing microhardness indentations at 50, 100, and 150 µm (white arrows) from the external enamel surface. Micro-Raman spectroscopy and EDX were performed at the same points.

A specimen from each treatment group was subjected to micro-Raman spectroscopy (RAMANtouch; Nanophoton Co., Ltd., Osaka, Japan) to detect the mineral composition at the same three beforementioned points. The excitation light used was a green laser beam with a wavelength of 532 nm in the spectral range of 400 to 1200 cm^−1^. Micro-Raman spectra were obtained using a × 50 objective to focus the laser beam on previously defined indentation points (Fig. 2). Origin version 6.1 (OriginLap, Northampton, MA, USA) and Peakfit version 4.0 (Aspire Software International, Ashburn, VA, USA) were used to analyze Raman spectra. The ratio of v_1_ PO_4_^3−^ (960 cm^−1^), v_2_ PO_4_^3−^ (431 cm^−1^), and v_4_ PO_4_^3−^ (589 cm^−1^) and B-type carbonate v_1_ CO_3_^2−^ (1072 cm^−1^) was obtained, but v_1_ PO_4_^3−^ (960 cm^−1^) and B-type carbonate v_1_CO_3_^2−^ (1072 cm^−1^) were generalized to analyze the change in mineral composition among the specimens.

### Elemental analysis using energy-dispersive X-ray spectroscopy (EDX)

**Figure 3:**
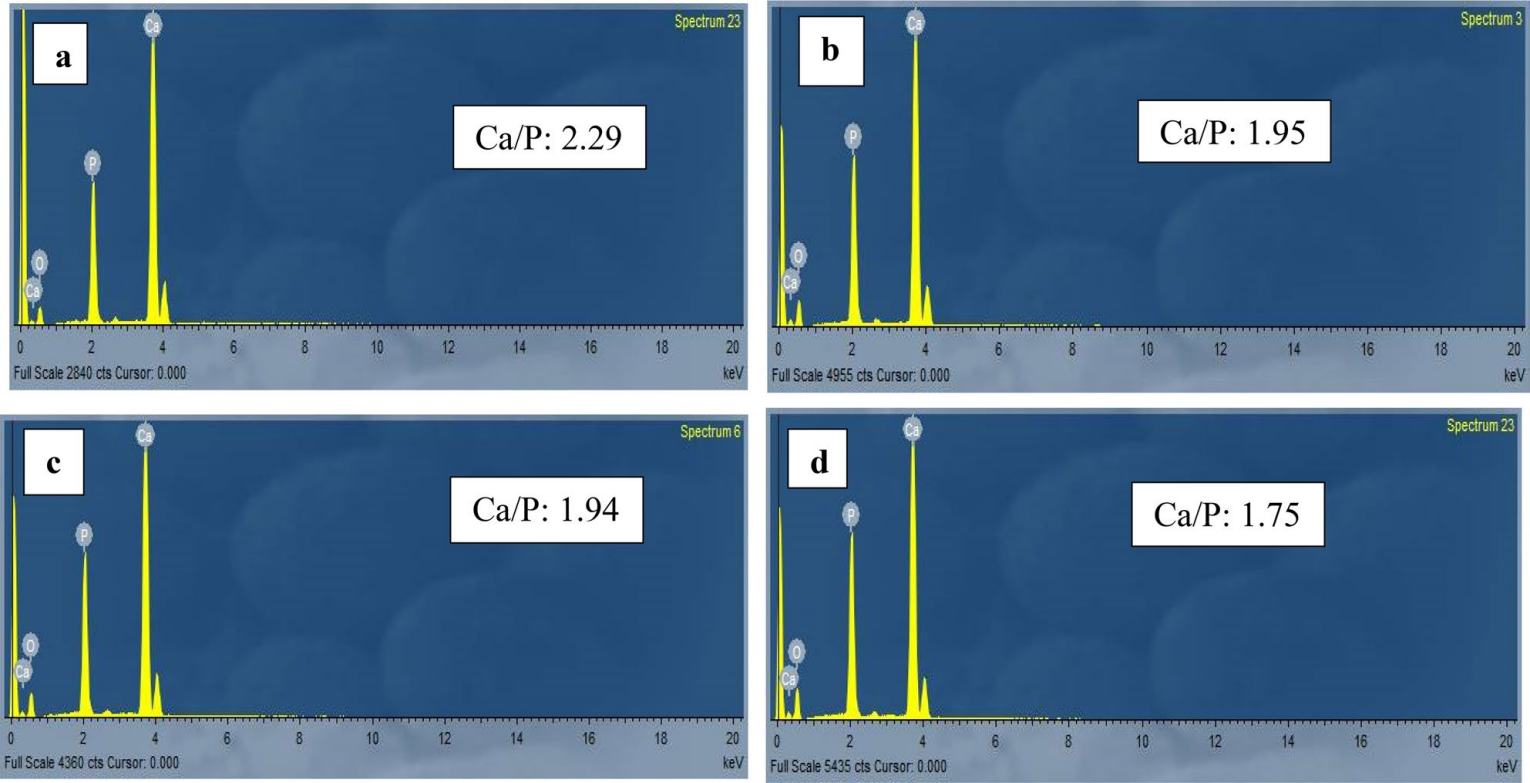
EDX of the remineralized specimen showing the Ca/P ratio at 150 µm: (**a**) prepared TCS, (**b**) CPP-ACP, (**c**) SDF-KI, and (**d**) control specimen.

The specimens were removed from acrylic blocks and coated with gold sputter to be evaluated with high vacuum field emission microscopy. The images were obtained using a secondary electron detector (Everhart–Thornley) at a distance of 10 mm with a high voltage of 30 kV and magnification ranging from × 500 to × 10,000. The EDS spectrometer was coupled with a scanning electron microscope (SEM; JEOL Ltd., USA) to quantitatively analyze the enamel mineral content. The EDX detector represented the calcium and phosphate weight percentages in a histogram plot (Fig. 3). The specimens were subjected to elemental analysis by EDX at the same three points (Fig. 2). Finally, micromorphological scanning with SEM at the superficial buccal surface (window site) was done.

### Statistical analysis

A sample size calculation with an estimated effect size of 0.60, an α of 5%, and a power of 85% resulted in a total sample size of 30 (n = 10 per group). The SPSS program (IBM SPSS Statistics, IBM, Armonk, NY, USA) was used to calculate descriptive statistics. Statistical analysis of the data was done, testing the normality using the Kolmogorov–Smirnov test applied before conducting the parametric tests [analysis of variance (ANOVA) and Tukey’s post hoc test]. Statistical significance was predetermined at p < 0.05.

### Ethical approval

The local Institutional Ethical Committee granted etichal approval for the current study under approval number (M03060819). All prosedures performed in the study were in accordance with 1964 Helsinki Declaration and its later ammendments or comparable ethical standards.

### Clinical relevance

The tricalcium silicate paste used in this study is a promising remineralizing agent for treating incipient lesions.

## Results

### CSMH results

In the three-way ANOVA, there was no significant difference in the remineralization potential among the different groups (p > 0.05). The mean ± standard deviation (SD) of the demineralized and remineralized specimens in the three different groups at three different levels (50, 100, and 150 µm) are shown in Table 2.

**Table 2:** Mean ± SD of CSMH of the different groups at different levels.

Material	Specimens	n	Microhardness (mean ± SD)		
			50 µm	100 µm	150 µm
SDF-KI	Demineralized	10	216.60 ± 46.22	244.90 ± 52.24^A^	261.40 ± 48.62^A^
	Remineralized	10	260.70 ± 21.87^ab^	288.20 ± 19.79^ac^	299 ± 19.86^bc^
CPP-ACP	Demineralized	10	190 ± 51.66	216.30 ± 55.87^A^	226.90 ± 50.55
	Remineralized	10	255.60 ± 45.11^a^	283.40 ± 45.19	297.7 ± 40.40^a^
TCS	Demineralized	10	173.60 ± 59.20	191.20 ± 63.67	209.2 ± 63.07^A^
	Remineralized	10	268.80 ± 29.23^a^	293.10 ± 29.23	307.60 ± 30.68^a^

^ABC^Similar superscript capital letters in the same column denote a significant difference among different materials within the same level on the same side (demineralized/remineralized; p < 0.05).

^abc^Similar superscript lowercase letters in the same row denote a significant difference among different levels within the same material on the same side (demineralized/remineralized; p < 0.05).

^$^Non-significant difference between the demineralized and remineralized specimens for the same level in the same material.

The mean ± SD and Tukey’s post hoc test multiple comparisons revealed a significant difference between the demineralized and remineralized specimens in the three different groups at the three different levels (50, 100, and 150 µm; p < 0.05). This reflected the remineralization power of the three tested materials. There was no significant difference among the specimens remineralized with SDF-KI, CPP-ACP, and TCS at the three different levels. There was a significant difference among the enamel microhardness at 50 and 150 µm in the three remineralized groups, reflecting the penetration power of the three tested materials.

There was a significant difference between demineralized specimens in the CPP-ACP and SDF-KI groups at 100 µm and the TCS and SDF-KI groups at 150 µm. Such discrepancies among demineralized specimens can be attributed to the following—the examined sound molars were collected from different patients of different ages. Age changes in old patients may compromise the hardness and mineral content of the sound enamel. Also, some of the examined molars were impacted molars, meaning that they had limited exposure to intraoral acidic cycles.

### EDX results

In the three-way ANOVA, there was a significant difference in Ca/P ratio among the different groups (p < 0.05). The mean ± standard deviation (SD) of the demineralized and remineralized specimens in the three different groups at three different levels (50, 100, and 150 µm) are shown in Table 3.

**Table 3:** Mean ± SD of Ca/P ratio of different groups at different levels.

Treatment material	Specimens	n	Ca/P ratio (mean ± SD)		
			50 µm	100 µm	150 µm
SDF-KI	Demineralized	10	1.69 ± 0.21	1.78 ± 0.21	1.84 ± 0.37^$^
	Remineralized	10	1.97 ± 0.12^A^	1.95 ± 0.07^A^	1.92 ± 0.12^B^
CPP-ACP	Demineralized	10	1.63 ± 0.18	1.73 ± 0.20	1.79 ± 0.34^$^
	Remineralized	10	1.96 ± 0.13^B^	2.00 ± 0.11	1.99 ± 0.14
TCS	Demineralized	10	1.63 ± 0.12	1.66 ± 0.102	1.67 ± 0.14
	Remineralized	10	2.09 ± 0.13^AB^	2.09 ± 0.155^A^	2.09 ± 0.16^B^

^ABC^ Similar superscript capital letters in the same column denote a significant difference among different materials within the same level on the same side (demineralized/remineralized).

^abc^ Similar superscript lowercase letters in the same row denote a significant difference among different levels within the same material on the same side (demineralized/remineralized).

^$^Nonsignificant difference between demineralized and remineralized sides for the same concentration in the same material.

The mean ± SD and Tukey’s post hoc test revealed a significant difference in the Ca/P ratio between the demineralized and remineralized specimens in the TCS group at the three different levels (p < 0.05).

Regarding CPP-ACP and SDF-KI, the significant difference in the Ca/P ratio between the demineralized and remineralized specimens was recorded at 50 and 100 µm (p < 0.05). At 150 µm, there was no significant difference between the demineralized and remineralized specimens (p > 0.05).

Also, there was a significant difference among the remineralized specimens in the three groups at 50 µm (p < 0.05). There was also a significant difference between the specimens remineralized with SDF-KI and TCS at 100 and 150 µm (p < 0.05). Prepared TCS showed the highest Ca/P ratio.

**Figure 4:**
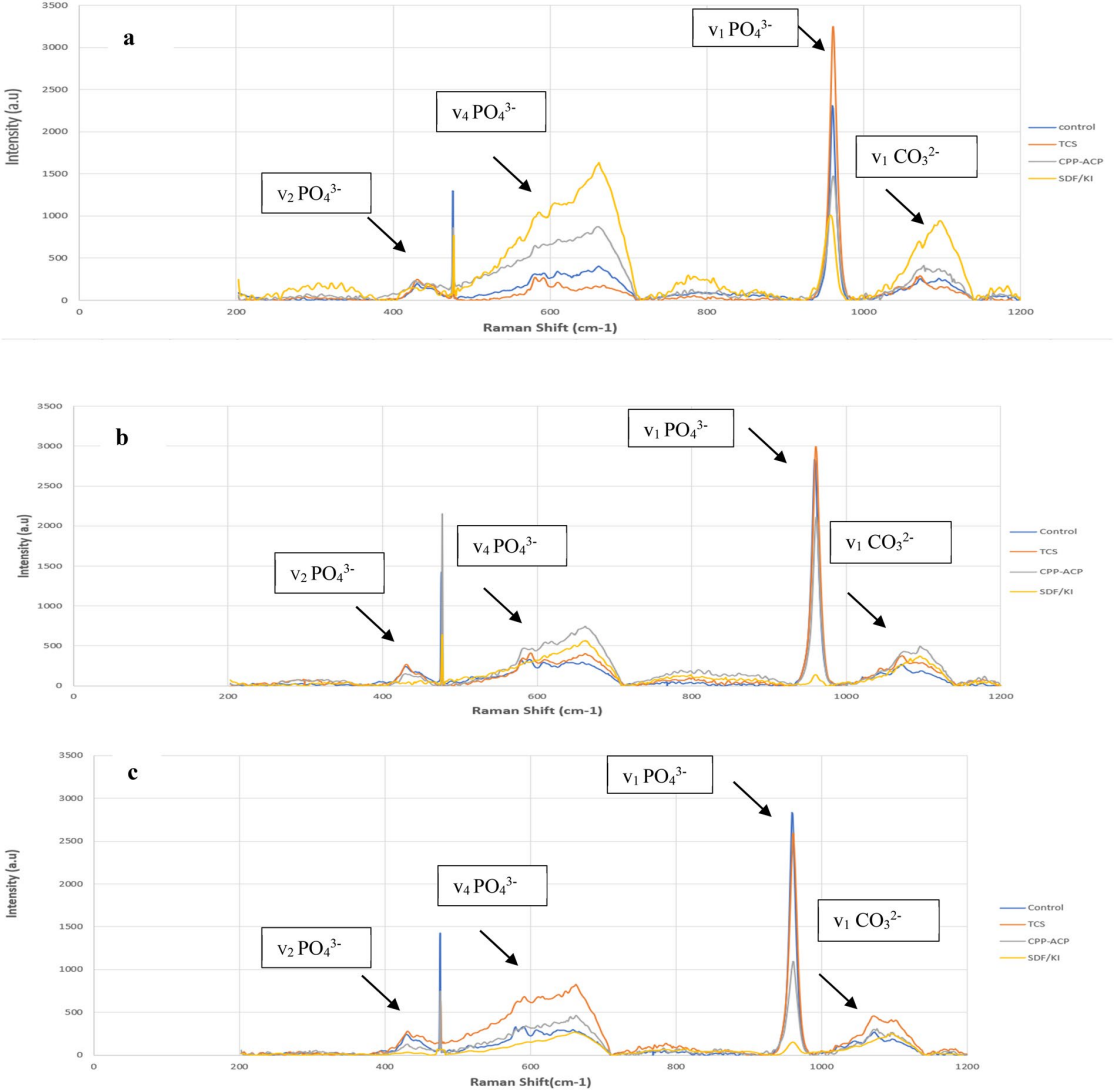
Micro-Raman spectroscopy of remineralized specimens showing the intensity of phosphate (v_1_ PO_4_^3−^) and carbonate (v_1_ CO_3_^2−^) bands at three different levels: (**a**) 50 µm, (**b**) 100 µm, and (**c**) 150 µm.

The major bands or parameters that can describe the mineral composition through micro-Raman spectroscopy corresponded to the phosphate (PO_4_^3−^) and carbonate (CO_3_^2^) groups. The phosphate group was associated with HAp crystals and had different vibration frequencies. The major vibration frequency of v_1_ PO_4_^3−^ was near 960 cm^−1^, whereas other minor vibration frequencies of v_2_ and v_4_ PO_4_^3−^ vibrations were detected near 431 and 589 cm^−1^, respectively. The vibration of v_1_ CO_3_^2−^ (B-type substitute) was detected near 1072 cm^−1^^22^. The intensity of bands assigned to CO_3_^2−^ reflected the carbonate content in the enamel and their substitution, which may be A- or B-type substitution. When the OH^−^ group is substituted by the CO_3_^2-^ group, this is defined as an A-type substitution, whereas a B-type substitution is expressed when the PO_4_^3−^ group is substituted by a CO_3_^2−^ one. The carbonate content revealed sufficient information about enamel hardness^22^. The increase in the intensity of the v_1_ CO_3_^2−^ band is an indicator of reduction in enamel hardness and its higher susceptibility to dissolution during acidogenic attacks. The high intensity of the v_1_ PO_4_^3−^ band indicated a high level of enamel mineralization^22^. All spectra were normalized based on the major vibration frequency v_1_ PO_4_^3−^, as it showed the most intense peak (Fig. 4). Specimens treated with TCS showed the highest intensity of v_1_ PO_4_^3−^ at the three different levels, whereas specimens treated with SDF-KI showed the lowest band intensity. SDF-KI also showed the highest intensity of the v_1_ CO_3_^2−^ band at 50 µm, reflecting the lowest remineralization power compared to CPP-ACP and TCS.

### Micromorphological analysis

**Figure 5:**
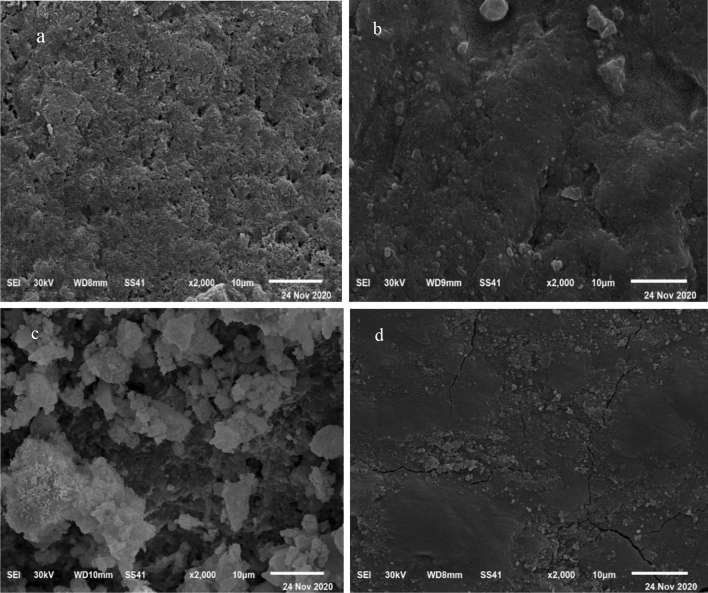
(**a**) SEM micrograph of a demineralized enamel surface. (**b**) SEM micrograph of an enamel substrate treated with CPP-ACP. (**c**) SEM of an enamel substrate treated with SDF-KI. (**d**) SEM micrograph of an enamel substrate treated with prepared TCS.

SEM of demineralized (self-control) specimens at high magnification (× 2000) showed observable pitting, discontinuity, and surface irregularity resulting from the destruction of enamel rods and the dissolution of enamel crystals during the demineralization process (Fig. 5a).

In the enamel substrate treated with CPP-ACP, SEM indicated the ability of CPP-ACP to restore the uniform, thick, and compact layer of disaggregated nanoclusters with a globular structure that decreased the size of interprismatic cavities (Fig. 5b).

SEM of enamel substrate treated with SDF-KI showed the ability of the SDF-KI solution to infiltrate the demineralized enamel, forming dense non-uniform crystals that well-sealed enamel interprismatic cavities (Fig. 5c).

SEM of the enamel substrate treated with experimental TCS indicated the ability of TCS paste to restore the uniform, compact, and homogenous layer of calcific deposits that well-sealed interprismatic cavities. The enamel substrate appeared with a low level of superficial roughness (Fig. 5d).

## Discussion

The null hypothesis of this study was partially accepted. The microhardness test revealed no significant difference in the remineralization potential of the three different materials at the three different levels (50, 100, and 150 µm). The EDX test revealed a significant difference among the three different materials at three levels.

The microhardness test is one of the most widely used tests to measure the mechanical properties and structural integrity of materials or substrates. Such an easy, simple, and nondestructive approach requires a limited specimen area to be tested. The specimen surface is impressed with a square-diamond indentation at a certain load for a certain period. After removing the load, the diagonal imprint is measured using an optical microscope to determine the length of the diagonal and size of the imprint^23^.

EDX is a quantitative X-ray microanalytical technique that provides sufficient information about the chemical composition of the targeted area of a sample. Such technology is based on the emission of a characteristic X-ray through a filament toward the sample^24^. An EDX detector can represent specific elements in a histogram plot with the number of counts against the X-ray energy^24^. Also, this powerful equipment combined with SEM magnifies and scans the area of interest and detects micromorphological changes^25^.

Schlueter et al.^26^ categorized SEM–EDX as a semiquantitative approach for elemental analysis. Cochrane et al.^27^ considered that the utilization of electron probe microanalysis generating X-ray in quantifying enamel mineral changes is a highly problematic approach. They addressed a list of drawbacks related to such technology, including non-uniform porosity that may cause density variations, an observed reduction of generated X-ray, and low interaction volumes for nonhomogeneous samples^27^. All these drawbacks justify using SEM–EDX in conjunction with another quantitative approach. The outcomes of this study are in line with Schlueter et al.’s^26^ and Cochrane et al.’s^27^ assumptions, as CSMH and SEM–EDX results did not totally confirm each other. Therefore, one specimen from each treatment group was subjected to micro-Raman spectroscopy. Raman spectroscopy is a quantitative, analytical, nondestructive approach that can detect the molecular level and composition by irradiating the specimen with a visible laser source^28^. Micro-Raman spectroscopy revealed that the most intensive v_1_ PO_4_^3−^ band was detected in the remineralized specimen with TCS at three levels (50, 100, and 150 µm). Specimens treated with CPP-ACP and SDF-KI showed the weak intensity of the v_1_ PO_4_^3−^ band.

Recently, the professional application of SDF-KI has gained wide acceptance due to its safety and effectiveness in either arresting or remineralizing early enamel surface lesions. Although sodium fluoride varnish is considered the gold standard protocol for enamel remineralization, SDF has shown comparable remineralization potential^29^. An in vitro study revealed the role of KI application to SDF in reducing the black staining effect and increasing enamel microhardness. SDF-KI showed potential rehardening of demineralized enamel than SDF alone^30^. According to published evidenc SDF-KI was incroporated in the current study as a representative of fluoride-based remineralizing products. Punhagui et al.^9^ concluded that the application of 38% SDF or even 30% SDF has a significant remineralization effect on the enamel surface and CSMH. Punhagui et al.^9^ conducted the enamel CSMH test at 10, 20, 50, 70, and 90 µm on deciduous enamel. However, the study is in line with Punhagui et al., citing that Punhagui et al.’s study may be considered weak evidence. Deciduous teeth have different pore sizes and mineral content than permanent teeth^31^. Also, this study is in total agreement with Barrera Ortega et al.’s^32^ study, which was conducted on permanent molars like this one. Barrera Ortega et al.^32^ evaluated the remineralization power of SDF by performing the CSMH test. They assumed that SDF increased the enamel microhardness up to 150 µm, in line with this study. The remineralization power of SDF was brilliantly explained by Mei et al.^33^. Mei et al.^33^ demonstrated that SDF could react with a salivary component, such as calcium and phosphate ions, forming fluorapatite crystals. These crystals can act as a nucleation site for further fluorapatite precipitates. Also, such crystals can promote ion exchange of F^−^ for OH^−^. Mei et al.^33^ also revealed an increase in the vibration frequency of phosphate peaks using Raman spectra, reflecting OH^−^ substitution with further F^−^ ions.

CPP-ACP has shown a satisfactory remineralization effect in different studies, and such an effect has been discussed previously in several cariology studies^34,35^. CPP contains Ser(P)-Ser(P)-Ser(P)-Glu-Glu sequence that can stabilize calcium, phosphate, and hydroxide ions as an amorphous nanocomplex (ACP), preventing rapid phase transformation^35^. Likewise, CPP-ACP can be localized on tooth surface buffering free calcium and phosphate ions, maintaining a state of supersaturation^35^. This study evaluated the remineralization potential of CPP-ACP on enamel subsurface lesions. This study agrees with previous similar studies^13,36^. Oliviera et al.^13^ concluded that MI paste could significantly reduce the demineralization effect on the outer enamel surface. Their CSMH indentations were conducted at 25, 50, 75, 100, 125, 150, 175, 200, 225, and 250 µm from the external enamel surface. They assumed that the MI paste showed a rehardening effect on the enamel subsurface up to 75 µm. This seems to be in partial agreement with this study. The slight difference in the findings between both may be ascribed to the length of each experiment. The length of Oliviera et al.’s study was 10 days, whereas the length of this study was 30 days. Also, CPP-ACP was applied once daily in Olivera et al.’s study, whereas it was applied twice daily in this study, justifying the slight difference between the findings. Another in vitro study showed the opposite results to this study^37^. The in vitro study demonstrated that CPP-ACP failed to remineralize the artificially created enamel subsurface lesion at 150 µm^37^. Such contradiction in results was attributable to the difference in the length and design of each study. The remineralization protocol of the in vitro study was based on applying a remineralizing agent, followed by pH cycling for 5 consecutive days only. Overall, the study was in accordance with Reynolds et al.’s assumption about the remineralization of subsurface enamel lesions using CPP-ACP^38^. Reynolds et al.^38^ highlighted the ability of the CPP-ACP nanocomplex to tightly bind to the enamel substrate, providing a reservoir of bioavailable calcium and phosphate ions. Microradiographs also recorded the ability of such bioavailable minerals to remineralize the body of the lesion^38^. The EDX revealed a higher Ca/P ratio of remineralized tissue than in normal HAp, in accordance with this study^38^.

Calcium silicate-based materials have an expanded range in restorative dentistry. One of the biggest advantages of these materials is their bioactivity, meaning that the material can react with body fluids producing mineral infiltration and deposition. Regarding enamel remineralization, there is little evidence suggesting using calcium silicate-based material in enamel remineralization^15^. An in vitro study demonstrated that when TCS (Ca_3_Sio_4_) comes in contact with saliva, it dissolves, forming a silanol group (si–o) that induces the precipitation of HAp^15^. Such a silanol group is characterized by obtaining triple junctions per unit area, which can provide a stereochemical match for oxygen atoms that will bond to Ca^2+^ ions^15^. Negatively charged PO_4_^3−^ ions in human saliva can be electrostatically attracted to positively charged Ca^2+^ ions, forming a dense Ca–P mineralized layer. An in vitro study also demonstrated the remineralization power of TCS in a slurry formula on the demineralized enamel^16^.

Parker et al.^39^ evaluated the repairable and protective effects of calcium silicate on sound and eroded enamel. Parker et al. assumed that micro-Raman spectra obtained from calcium silicate showed characteristic peaks similar to HAp^39^. Other in vitro and in situ experiments identified HAp formation on the enamel surface after brushing with a slurry of calcium silicate^40^. In vitro studies that evaluated the remineralization potential of TCS used it in the slurry formula. The clinical application of TCS in slurry form may be very difficult for patients. Furthermore, TCS formulation in such a manner may be time- and material-consuming each time. Therefore, in this study, TCS was prepared in a paste form for easy application in future clinical research if it showed promising results. Also, TCS in a paste formula can be tightly bound to the enamel substrate, providing biofilm supersaturation with minerals, maximizing its remineralization potential. Such formula was inspired by similar bioactive remineralizing products with the same remineralization rationale, such as Novamin paste and 45S5 Bioactive glass^41–43^. Generally speaking, regarding the remineralization power of TCS, the discussed evidence in the literature agrees with this study. Although several biological components, such as proteins and extracellular matrix, are involved in enamel mineralization and crystal nucleation, TCS showed potential remineralization that may compel community research to investigate further. In this study, specimens treated with TCS showed promising rehardening of the softened enamel up to 150 µm. Also, EDX results showed a superior remineralization power of TCS on subsurface enamel lesions up to 150 µm compared to CPP-ACP and SDF-KI. Micro-Raman spectroscopy also supported EDX findings, as the highest v_1_ PO_4_^3−^ band intensity was observed in the specimen treated with TCS at three levels. Therefore, TCS is superior in remineralization and penetration ability in artificial enamel lesions.

Human saliva plays a crucial role in enamel remineralization, as it contains organic components (e.g., glycoproteins) and inorganic components (e.g., calcium, phosphate, and fluoride). When the human saliva gets out of the oral environment, its protective effect may be compromised due to changes in its composition ^20^. Collecting human saliva is also very difficult and time-consuming. Due to the limitations of using natural saliva in in vitro studies, substitutive formulations must be available to simulate the oral environment in remineralization studies^20^. Different formulations of artificial saliva are available for remineralization studies. There is no consensus in cariology research on the ideal formula to be used in remineralization studies^20^. The used formula in this study had shown the lowest remineralization power compared to four different formulas^20^. The formula of the used artificial saliva in this study contains carboxymethyl cellulose. CMC increases the viscosity of the artificial saliva solution, consequently limiting the mineral diffusion to the enamel substrate^20^. Also, CMC can form complexes between calcium and phosphate ions, resulting in the unavailability of such minerals in enamel remineralization^20^. The rationale behind selecting such a formula is to provide some sort of accurate evaluation of tested materials and avoid generating misleading results.

The outcomes of the current study are limited by comparing tricalcium silicate paste with single material (CPP-ACP) that belongs to the same category (bioavailable calcium phosphate remineralizing system). It would be better if another bioactive material were included in the study. This will be considered in the future studies.

## Conclusion

In this study, prepared TCS paste was a powerful remineralizing agent for managing early enamel lesions. Prepared TCS showed that the remineralization power of enamel subsurface lesions reached up to 150 µm. This approach seems to be a safe and effective remineralization approach for managing early enamel carious lesions.

## Supplementary Information

Supplementary Information 1.

Supplementary Information 2.

Supplementary Information 3.

Supplementary Information 4.

Supplementary Information 5.

## Data Availability

The data that support the findings of the current study are available from corresponding author upon resonable request.
